# Cooked Red Lentils Dose-Dependently Modulate the Colonic Microenvironment in Healthy C57Bl/6 Male Mice

**DOI:** 10.3390/nu11081853

**Published:** 2019-08-09

**Authors:** Daniela Graf, Jennifer M. Monk, Dion Lepp, Wenqing Wu, Laurel McGillis, Kyle Roberton, Yolanda Brummer, Susan M. Tosh, Krista A. Power

**Affiliations:** 1 Guelph Research and Development Center, Agriculture and Agri-Food Canada, Guelph, ON N1G 5C9, Canada; 2 Human Health and Nutritional Sciences, University of Guelph, Guelph, ON N1G 2W1, Canada; 3 Department of Physiology and Biochemistry of Nutrition, Max Rubner-Institute, 76131 Karlsruhe, Germany; 4 School of Nutrition Sciences, University of Ottawa, Ottawa, ON K1H 8L1, Canada

**Keywords:** Red lentils, microbiota, colon, short-chain fatty acids, pectin

## Abstract

Dietary pulses, including lentils, are protein-rich plant foods that are enriched in intestinal health-promoting bioactives, such as non-digestible carbohydrates and phenolic compounds. The aim of this study was to investigate the effect of diets supplemented with cooked red lentils on the colonic microenvironment (microbiota composition and activity and epithelial barrier integrity and function). C57Bl/6 male mice were fed one of five diets: a control basal diet (BD), a BD-supplemented diet with 5, 10 or 20% cooked red lentils (by weight), or a BD-supplemented diet with 0.7% pectin (equivalent soluble fiber level as found in the 20% lentil diet). Red lentil supplementation resulted in increased: (1) fecal microbiota α-diversity; (2) abundance of short-chain fatty acid (SCFA)-producing bacteria (e.g., *Prevotella, Roseburia* and *Dorea spp.*); (3) concentrations of fecal SCFAs; (4) mRNA expression of SCFA receptors (G-protein-coupled receptors (*GPR 41 and 43*) and tight/adherens junction proteins (Zona Occulden-1 (*ZO-1*), Claudin-2, E-cadherin). Overall, 20% lentil had the greatest impact on colon health outcomes, which were in part explained by a change in the soluble and insoluble fiber profile of the diet. These results support recent public health recommendations to increase consumption of plant-based protein foods for improved health, in particular intestinal health.

## 1. Introduction

Dietary pulses, including lentils (*Lens culinaris*), common beans (*Phaseolus vulgaris*), chickpeas (*Cicer arietinum*), and peas (*Pisum sativum*), have been part of the human diet for more than 10,000 years [1]. Pulses are considered highly nutritious foods due to their high content of protein (20–25%) [2], non-digestible carbohydrates (e.g., soluble and insoluble fibers, resistant starch, galacto-oligosaccharides (GOS)) (14–32%) [3], vitamins and minerals (2–5%) [4], and phenolic compounds (~1%) [5,6], although the nutritional composition varies between different pulse types and across pulse varieties [7,8]. Pulse consumption has been associated with a reduced risk of chronic diseases, including coronary heart disease, diabetes, and colorectal cancer [9,10,11,12]. However, worldwide pulse consumption has decreased in recent decades [13]. In Canada, only 13% of the population consume pulses on any given day, with a mean intake of approximately 110 g/day (~1/2 cup/day) [13]. Therefore, research efforts to demonstrate and elucidate the mechanisms through which pulses improve aspects of human health may help to enhance pulse consumption. This is of increasing importance as the recent launch of the new Canada’s Food Guide promotes increased consumption of protein-rich plant foods, which includes dietary pulses [14]. 

Due to the diverse content of non-digestible carbohydrates and phenolic compounds, our group and others have studied the potential for pulse foods to modulate intestinal health [15,16,17,18] and intestine related-diseases, including Inflammatory Bowel Disease (IBD) [17,19,20,21,22], colon cancer [23,24,25,26,27], obesity [28,29,30,31], and Type-2 Diabetes [32,33,34]. Importantly, these diseases are associated with microbial dysbiosis, intestinal barrier dysfunction, and intestinal and systemic inflammation, and thus, dietary modulation of components of the intestinal microenvironment may prove beneficial in preventing and/or attenuating disease development and severity [35,36,37]. In healthy mice, we have demonstrated that dietary supplementation with cooked chickpeas and different common bean varieties exert beneficial priming effects on the colonic epithelium including an increase in goblet cell density, mucin production, and upregulation of mediators that promote colon barrier integrity and function (e.g., tight junction proteins, anti-microbial peptides, microbial defense response proteins) [15,16,17,21,22]. Further, pulse-supplemented diets can beneficially modulate the murine intestinal microbiota composition and activity, leading to the enhanced production of microbial-derived short-chain fatty acids (SCFAs) [15,16,17,21,22] including acetate, propionate, and butyrate, and enrichment of SCFA-producing microbiota, including *Prevotella spp., Ruminococcus flavefaciens*, and *S24-7* [15,16,17]. These findings are also supported in *in vitro* fecal fermentation experiments showing that various pulse types are potent substrates for microbial fermentation and SCFA production [8,38,39]. SCFAs, in particular butyrate, promote intestinal health by providing energy for colonic epithelial cells and inhibiting histone deacetylases, thereby contributing to the anti-carcinogenic effects of SCFAs [40]. Furthermore, SCFAs modulate inflammation by inhibiting pro-inflammatory cytokine production and enhancing the activation of immunosuppressive regulatory T cells [37,41], and promote epithelial barrier integrity by increasing tight junction protein expression [42]. Our previous research has also demonstrated the anti-inflammatory and epithelial barrier promoting potential of pulse-supplemented diets in a mouse model using dextran sodium sulphate (DSS)-induced colitis, which may be driven, in part, through the production of SCFAs [17,19,20,22]. 

Lentils have received minimal attention concerning their potential to modulate intestinal health and disease [43,44,45]. Among the pulse food types, the incorporation of lentils into the North American diet may be more easily achieved due to their small seed size and shorter preparation (e.g., no soaking required) and cooking time [46,47,48]. However, in contrast to other pulses, in particular common beans, lentils contain lower levels of non-digestible carbohydrates, including total dietary fiber [49], resistant starch [50], soluble fiber and galacto-oligosaccharides (GOS) [49]. On the other hand, lentils are amongst the highest with regards to their phenolic compound content compared to other pulse types (e.g., peas and chickpeas), which contributes to their high antioxidant potential [51,52,53]. The phytochemicals found in lentils include oligomeric and polymeric proanthocyanidins (e.g., catechins) [54,55], kaempferol glycosides [56] and phenolic acids (e.g., chlorogenic acid) [57]) and carotenoids (especially luteins and zeaxanthins) [58]. Phenolic compounds and their microbial-derived secondary metabolites induce diverse effects on the intestinal microenvironment including altering the microbial community structure [59,60,61], enhancing intestinal barrier integrity [62,63], attenuating oxidative stress [53,54,64,65,66], and reducing colonic inflammation [66,67,68]. Consequently, like other pulses, lentils may induce beneficial effects on intestinal health through their fiber and phenolic compound components. However, due to the inherent variation in quantity and composition compared to that of other pulse types, the effects may differ. 

The aim of the present study was to investigate the effects of diets supplemented with cooked red lentils on the colonic microenvironment in disease-free male mice, including the fecal microbial community structure and activity, as well as the integrity and function of the mucosal barrier. The impact of different physiologically relevant lentil supplementation levels (5, 10 and 20% (by weight)) was investigated in order to establish the consumption levels required for significant intestinal health outcomes. To determine whether the intestinal health effects induced by lentil supplementation were attributable to fermentable soluble fiber, an additional group was included which received the BD supplemented with the same concentration of pectin (most abundant class of soluble non-starch polysaccharides found in lentils [50]) equivalent to the soluble fiber content in the 20% lentil diet.

## 2. Materials and Methods

### 2.1. Preparation of Red Lentil Flour and Experimental Diets

Red Lentils (CDC Maxim cultivar) were rinsed in cold water and 200 g batches were cooked with 500 mL deionized water in a slow cooker. After lentils were brought to a boil, they were allowed to simmer for 30 minutes. Cooked lentils and cook water were cooled, blended into a puree, freeze-dried, and sifted through a brass wire 129 sieve with a 1 mm pore size (VWR, Mississauga, ON, Canada) to produce a cooked red lentil powder with a uniform particle size. Red lentil powder macronutrient and fiber content was measured by Maxxam Analytics (Mississauga, Ontario, Canada) and reported as (% by weight): protein, 27.6; available carbohydrates, 47.9; fat, 1.6; ash, 2.7; moisture, 2.6; total fiber (insoluble + soluble), 17.6; insoluble fiber, 14.3; and soluble fiber, 3.4 (Association of Official Analytical Chemists (AOAC) Method 994.43). The pectin content of the cooked lentil powder was analyzed by measuring the anhydrogalacturonic acid content using the spectrophotometric method of Blumenkranz and Asboe-Hansen [69] after solubilization in sulfuric acid, and was found to be 2.43% (by weight). This confirms that the majority of the lentil flour soluble fiber fraction (3.4% by weight as measured by Maxxam Analytics (Mississauga, Ontario, Canada)) is comprised of pectic substances, as previously reported [50]. The GOS content of the cooked lentil powder was determined by high pressure anion exchange chromatography with pulsed amperometric detection (DX600, Thermo Scientific Dionex, Sunnyvale, CA) as previously reported [45] and found to be 3.53 ± 0.06g/100 g on a dry weight basis. The composition of the GOS fraction was 7% raffinose, 65% stachyose and 29% verbascose. GOS are not measured in the AOAC Method 994.43 and can be considered a fermentable carbohydrate source for the microbiota, in addition to the soluble fiber reported. Resistant starch in lentil powder was measured using AOAC Method 2002.02 and determined to be 4.3 ± 0.2g/100 g on a dry weight basis. Resistant starch may be partially measured in the AOAC 994.43 dietary fiber method and may account for part of the soluble fiber fraction reported. 

Five isocaloric diets with matching macronutrient content were prepared by Envigo (Tekland; Madison, WI, USA) (Table 1) including a modified American Institute of Nutrition (AIN)-93G basal diet (BD) (7% soybean oil substituted with 7% corn oil, and the cellulose content increased from 5 to 7%); a BD-supplemented diet with 5, 10 or 20% lentil powder (*wt*/*wt*) (LD); and a mixed-fiber control diet (pectin) prepared by supplementing the BD with 0.7% soluble fiber (citrus pectin; Sigma Aldrich (P9135)) to replace part of the cellulose (insoluble fiber), which achieves an equivalent level and ratio of insoluble to soluble fiber present in the 20% LD. This mixed-fiber control group was included to determine whether the effects induced by lentils were solely due to a change in non-digestible carbohydrate (fiber) profile.

### 2.2. Study Design

All experimental procedures were approved by the institutional animal care committee (University of Guelph; animal use protocol #3115) in accordance with the Canadian Council of Animal Care. A total of 60 5wk-old male C57Bl/6 mice were purchased from Charles River (Kingston, NY, USA), housed 3 mice/cage, and given free access to BD and water, as previously described [21]. After one week of acclimation, mice were randomly assigned to five groups (*n* = 12/group) and given their respective experimental diets ad libitum for three weeks. Body weight (BW) and diet intake were assessed twice weekly. Fresh feces were collected prior to sacrifice under sterile conditions, snap frozen in liquid nitrogen (LqN2), and stored at −80 °C until further use. At the end of the study, mice received an intraperitoneal injection of 30 µg/g BW 5-ethynyl-2’-deoxyuridine (EdU) and were then euthanized two hours later by cervical dislocation. Colons were excised from the ceco-colonic junction to the rectum and a 1-cm piece of proximal colon containing a fecal pellet was fixed in Carnoy’s fixative for analysis of mucus layer thickness; an empty 1-cm piece of proximal and distal colon were fixed in 10% buffered formalin solution for histological analysis, and the remaining colon was snap frozen in lqN2 and stored at −80°C for later mRNA expression analyses. 

### 2.3. Fecal Microbiome Analyses

Genomic DNA was extracted from fecal samples (*n* = 9 mice/group) using the QiaAmp Fast DNA stool mini kit (Qiagen, Valencia, CA, USA). Sequencing libraries of the 16S V3-4 region were prepared according to the Illumina 16S Metagenomic Sequencing Library Preparation Guide Rev. B as described previously [17,70]. Sequencing reaction amplification and purification was performed exactly as described [17,70] using 12.5 ng of template DNA, 200 nM each primer and 1X KAPA HiFi HotStart ReadyMix (VWR, Mississauga, ON, Canada) and Ampure XP beads (Beckman Coulter, Mississauga, ON, Canada). Sequencing adapters containing 8-bp indices were added to the 3’ and 5’ ends by PCR using the Nextera XT Index kit (Illumina, San Diego, CA, USA) followed by a second purification with Ampure XP beads as described [17,70]. Amplicons were quantified using the Quant-iT PicoGreen double-stranded DNA assay (Invitrogen/Life Technologies Inc., Burlington, ON, Canada) and equimolar ratios were pooled and combined with 5% equimolar PhiX DNA (Illumina) for sequencing on a MiSeq instrument, using the MiSeq 600-cycle v3 kit (Illumina).

The resulting sequence data containing 300-base pair (bp) dual-indexed paired-end reads were processed with QIIME v1.9.1 [71]. The paired-end reads were first joined by aligning overlapping sequences with fastq-join [72], and then quality filtered and demultiplexed in QIIME using default settings. The remaining reads were clustered at 97% identity with uclust [73] and operational taxonomic units (OTUs) were picked using an open-reference approach with the GreenGenes database (gg otus 13 8) [74] as a reference, and taxonomy was assigned with the uclust consensus taxonomy assigner. The sequences were aligned against the GreenGenes core set with PyNast [75] and a phylogenetic tree constructed with FastTree [76]. OTUs representing < 0.005% of the population were removed from the resulting OTU table. Alpha-diversity metrics were then calculated by QIIME using a read depth of 27,000 and a β-diversity distance matrix based on UniFrac metric [77] was calculated, which was used for principal coordinates analysis (PCoA). PCoA plots were generated with Phylotoast v 1.3.0 [78]. The significance of the diet effect on the β-diversity distance matrix was assessed by PERMANOVA analysis. Significant differences (*p* < 0.05; false discovery rate (FDR) < 0.1) in the relative abundance of taxa between the different diets were determined by the Kruskal–Wallis test followed by Dunn’s multiple comparisons test using the R package dunn.test version 1.3.2. Correlation analysis was calculated by Spearman’s correlation method in Qiime observation_metadata_correlation.py (*p* < 0.05; false discovery rate (FDR) < 0.05).

Phylogenetic Investigation of Communities by Reconstruction of Unobserved States (PICRUSt) v.1.0.0 [79] was used to infer metagenomes based on 16S marker data and predicted Kyoto Encyclopedia of Genes and Genomes (KEGG) pathway abundances. Statistical analysis and visualization of KEGG pathway data was performed with Statistical Analysis of Metagenomic Profiles (STAMP) v2.1.3 [80] using the Welsh’s t-test and the Benjamini–Hochberg procedure to control the FDR (< 0.02). Non-bacterial pathways may be predicted by PICRUSt due to inaccuracies in the underlying bacterial genome sequence annotations and, therefore, these pathways (i.e., endocrine systems, human diseases, etc.) were filtered from the analysis.

### 2.4. Fecal SCFA and Branched-Chain Fatty Acid (BCFA) Analyses

Gas chromatography was used to measure fecal SCFA (acetic acid, propionic acid, butyric acid and valeric acid) and BCFA concentrations (isobutyric acid and isovaleric), which are microbial metabolites of saccharolytic and proteolytic activity, respectively. A total of 20 mg of freeze-dried (Freezone 12, bulk tray dryer (Labconco, Toronto, Canada)) fecal samples were homogenized in 200 µL of MilliQ water and centrifuged at 14,000× *g* at 4 °C for 30 min. Supernatant pH was measured using Thermo Scientific™ Orion Star™ A111 pH Benchtop Meter, Thermo Scientific Ross Micro pH Electrode. A volume of 10 µL of 5.5 mmol/L 2-ethylbutyric acid (Sigma 109959, Oakville, ON, Canada), in formic acid, was added to 100 µL of the fecal supernatant as an internal standard. The pH was adjusted to 2–3 by adding formic acid, and centrifuged at 14000×g at 4 °C for 30 min. The samples (1 µL) were injected into a gas chromatography unit (Agilent 6890, Mississauga, ON, Canada), equipped with a flame ionization detector (FID) and a Nukol-capillary GC column (60 m × 0.25 mm × 0.25 µm, Sigma-24108 SUPELCO, Oakville, ON, Canada). Helium was used as the carrier gas; the initial oven temperature was 100 °C and was increased to 200 °C at a rate of 10 °C/min; injector and detector temperatures were maintained at 200 and 300 °C, respectively. The total running time was 20 min for each injection. The peaks were identified by comparing their retention times with Volatile acid standard mix (Sigma 46975-u, Oakville, ON, Canada). The data collection was managed using OpenLAB ChemStation software developed by Agilent Technologies, Canada. The concentration of fecal SCFA and BCFA were expressed as µmol/g of dry fecal weight. Each sample injection was repeated three times.

### 2.5. Colon Histology 

For histological analyses, colon tissues were fixed using Carnoy’s solution or formalin, embedded in paraffin, sectioned (5µm), and placed on glass slides. Hematoxylin and Eosin (H&E)-stained sections were analyzed for crypt length and number of goblet cells/crypts as previously described [15]. For crypt length, 15–20 crypts/mouse were assessed. To measure goblet cell density, the number of goblet cells/10–15 crypts/mouse were counted (*n* = 7–11/group). Additionally, colon crypt mucus content was measured in Alcian Blue/Nuclear Fast Red (AB)-stained colon cross sections as described in detail elsewhere [15]. Carnoy’s-fixed colon sections were stained with AB for measurement of mucus layer thickness. Average mucus layer thickness was obtained by taking 40 measurements within 4 fields at a 100x magnification per animal (*n* = 8–10/group). All images of H&E-and AB-stained colon sections were captured using a BX51 microscope (Olympus) with an Olympus DP72 digital camera system and the Image J software (National Institute of Health) was used for analysis.

### 2.6. Cell Proliferation

EdU incorporation into the DNA was analyzed using the ClickiT EdU Alexa Fluor 647 Imaging kit (Molecular Probes/Thermo Fisher Scientific) and the proliferation index of colonic epithelial cells was assessed as described previously [16]. For each mouse, two images were captured (100× magnification) on an Imager A2 microscope equipped with an AxioCamMRc5 camera system and Zen 2 (Blue Edition) software (Zeiss Canada Ltd., Toronto, ON, Canada). Fluorescence intensity was measure using Image J Software and the proliferation index was calculated according to the following formula: Proliferation Index = (fluorescence intensity Alexa Fluor 647/fluorescence intensity Hoechst) ⁄ 100.

### 2.7. Colonic mRNA Expression

Colon RNA was isolated using the RNA/protein Purification Plus Kit (Norgen Biotek, Thorold ON, Canada). Total RNA (2 μg) was converted to cDNA using the High Capacity cDNA Reverse Transcription kit (Applied Biosystems, Foster City, CA, USA) and the relative mRNA expression of target genes was assessed by quantitative real-time PCR using Power SYBR Green PCR Master Mix (Applied Biosystems) and the 7900HT Fast Real-Time PCR system (Life Technologies Inc., Burlington, ON, Canada). Data were analyzed using the 2^(40-CT)^ method and normalized to the expression of the housekeeping gene *RPLP0*. All primer sequences have been previously published [15,16]. 

### 2.8. Statistical Analysis

BW and diet intake were analyzed using a two-way repeated measures Analysis of variance (ANOVA) (main effects: diet and time). All other data were analyzed using one-way ANOVA or the Kruskal–Wallis Test, depending on distribution (D’Agostino and Pearson omnibus normality test) and Newman–Keuls or Dunn’s post-hoc test respectively. Differences were considered significant with *p* < 0.05 and data are presented as mean ± SEM. Data analysis and visualization was performed using GraphPad Prism 8.1. 

## 3. Results

### 3.1. Consumption of Lentil-Supplemented Diets Do Not Alter Food Intake or Body Weight Gain in C57Bl/6 Male Mice

**Figure 1:**
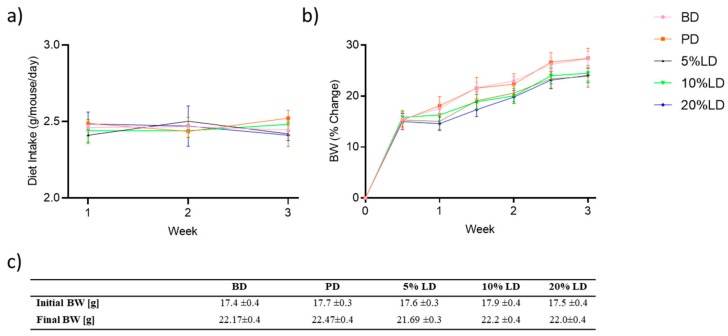
The effect of lentil supplementation on body weight and diet intake. (**a**) Average diet intake (g/mouse/day), (**b**) body weight (BW) change (%), and (**c**) initial and final BW (g) of C57Bl/6 mice receiving basal diet (BD) or diets supplemented with pectin (PD), or 5, 10, 20% lentils (LD). Values are mean ± SEM; *n* = 12/group; *p* > 0.05.

At randomization and at the end of the study, BW did not differ between the groups (Figure 1c). BW gain was monitored regularly throughout the study but did not differ at any time point between the groups (Figure 1b). Average daily food intake throughout the intervention period did not differ between groups (Figure 1a).

### 3.2. Consumption of Lentil-Supplemented Diets Modulate Fecal Microbial Community Structure and Activity

#### 3.2.1. Microbial Community Diversity

**Figure 2:**
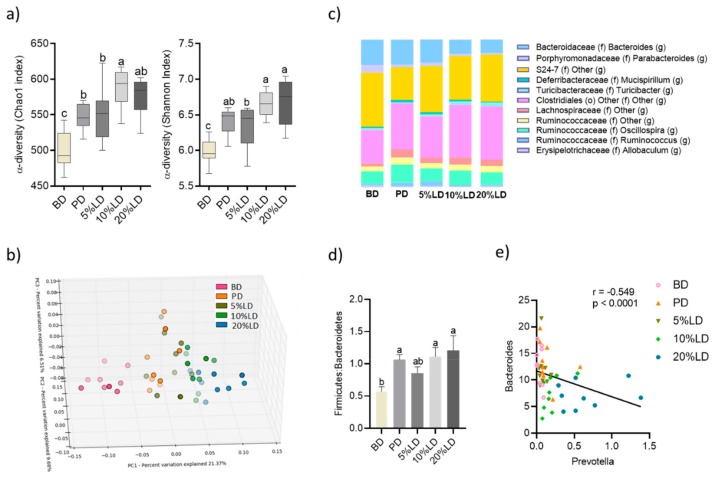
The effects of lentil supplementation on the fecal microbiota composition.(**a**) Fecal microbiota α-diversity as measured by Choa-1 and Shannon diversity indices; (**b**) β-diversity as shown by principal coordinate analysis (PCoA) of unweighted UniFrac distance matrices showing bacterial communities cluster within dietary groups and the percent of dataset variability explained by each principal coordinate is shown in the axis titles (Principle coordinate 1(PC1): 21.4%; PC2: 9.7%; PC3: 6.51%); Each dot represents one mouse and each group is denoted by a different colored symbol (pink: basal diet (BD); orange: pectin diet (PD); dark green: 5% lentil diet (LD); light green: 10% LD; blue: 20% LD); (**c**) microbiota taxa composition at the corresponding order (o), family (f) or genus (g) level (includes taxa representing >0.5% total composition in at least one sample); (**d**) Firmicutes/Bacteroidetes ratio; values are mean ± SEM; columns without a common letter differ significantly (*p* < 0.05); (**e**) correlation between *Prevotella* and *Bacteroides* abundance calculated by Spearman’s correlation method (*p* < 0.05; false discovery rate (FDR): 0.05); each symbol represents one mouse and each group is denoted by a different colored symbol (pink: BD; orange: PD; dark green: 5% LD; light green: 10% LD; blue: 20% LD).

To assess the impact of consuming diets supplemented with cooked lentils on the intestinal microbial community structure, fecal DNA was extracted, and microbial composition was analyzed using 16S rRNA gene sequencing, following three weeks of dietary intervention. Compared to the BD group, lentil supplementation increased fecal microbial α-diversity, as measured by Chao1 and Shannon Diversity indices (Figure 2a), with the 10 and 20% LD groups having higher α-diversity (Shannon Diversity indices) compared to the 5% lentil group, suggesting that the level of lentil supplementation was important in altering microbial diversity. In addition, the fecal α-diversity in the PD group was higher than BD, and generally did not differ from lentil groups. Microbial community β-diversity differed significantly between the five dietary intervention groups (*p* ≤ 0.001). Principal Coordinate Analyses (PCoA) of unweighted Unifrac distance matrices revealed clustering of samples according to diet, with the greatest apparent difference in community composition observed between the BD and 20% lentil group (Figure 2b).

#### 3.2.2. Microbial Community Structure

To investigate the underlying taxonomic differences between groups, we assessed fecal microbiota composition at different taxonomic levels and the phylum (Table 2) and genus level (Figure 2c; Table 2) are shown. In the BD group, as expected, Bacteroidetes (~60%) and Firmicutes (~33%) represented the dominant phyla in the fecal microbial community. Consumption of diets supplemented with 10 and 20% lentils increased the proportion of Firmicutes (from ~33 to ~50%), thereby increasing the Firmicutes/Bacteroidetes ratio (Figure 2d). This effect was less apparent in the 5% LD group, suggesting this effect may be dose dependent. Similar to the effects induced by 10 and 20% LD, mice fed pectin-supplemented diets enhanced Firmicutes abundance compared to BD (Table 2), thereby increasing the Firmicutes/Bacteroidetes ratio (Figure 2d), suggesting an impact of pectin fiber in the observed effects induced by lentils. Within minor phyla, LD enhanced the abundance of TM7, which was primarily due to an increase in bacteria from the F16 family. However, no difference was observed when comparing BD and PD groups, suggesting that other lentil components may be responsible for this effect (Table 2).

Within the Bacteroidetes phylum, 10 and 20% LD reduced *Bacteroides* abundance compared to the BD group, while 5% LD did not differ from BD (Table 2), suggesting this effect may be dose dependent. Furthermore, fecal *Bacteroides* abundance in mice fed the pectin-supplemented diet did not differ from BD, suggesting that the effect of lentils was not solely due to a change in dietary insoluble and soluble fiber profile. Fecal *Prevotella* abundance was increased by both 10 and 20% LD, but not by the 5% LD, with 20% LD inducing the greatest effect. In contrast, all LD groups exhibited reduced *Parabacteroides* abundance compared to BD. The effects of lentils on the abundance of *Prevotella* and *Parabacteroides* may be due in part to the presence of soluble fiber in the diet, since mice fed pectin-supplemented diets demonstrated similar changes (increased *Prevotella*; decreased *Parabacteroides*) compared to BD (Table 2). Interestingly, it has been shown that a diet high in animal-based products is associated with an increased abundance of *Bacteroides spp.*, whereas a diet high in plant foods and dietary fiber is enriched in members of the *Prevotella* genus, which play a role in the degradation of complex carbohydrates and production of SCFAs [81]. In line with this, we observed a negative correlation between the abundance of *Bacteroides* and *Prevotella* (Spearman *r* = −0.5487; *p* < 0.0001) which was driven by the increasing level of lentil in the diet (Figure 2e). 

The observed lentil-induced increase in Firmicutes was in part due to an unknown genus belonging to the *Lachnospiraceae* family and the genera *Coprococcus*, *Dorea*, and *Roseburia* **(**Table 2). Furthermore, 20% LD, but not 5 and 10% LD, significantly increased the genus *Turicibacter*. PD also increased the abundance of the unknown genus belonging to the *Lachnospiraceae* family and the genus *Coprococcus* compared to BD. However, in contrast to the supplementation with lentils, supplementation with pectin did not increase abundance of *Dorea*, *Roseburia* and *Turicibacter* indicating that lentil components other than its soluble pectin fraction, can modulate the fecal microbiota composition (Table 2).

Metagenomic changes between the BD and 20% LD groups were predicted and functionally annotated using PICRUSt, which identified 35 KEGG pathways that differed significantly between groups (13 features were enriched in the 20% LD group and 22 features were enriched in the BD group) (Figure S1). The metagenomes of the 20% LD group were enriched for genes related to the biosynthesis of other secondary metabolites (e.g., phenylproponoid, flavone and flavonol, and flavonoid biosysthesis), carbohydrate metabolism (e.g., inositol phosphate metabolism, ascorbate and aldarate metabolism, and butanoate metabolism), and metabolism of other amino acids (e.g., cyanoamino acid metabolism and D-arginine and D-ornithine metabolism). On the other hand, the BD group showed enrichment in identified KEGG pathways related to lipid metabolism (e.g., fatty acid biosynthesis, primary and secondary bile acid synthesis, and lipid biosynthesis proteins), immune system (e.g., nucleotide-binding oligomerization domain (NOD)-like receptor signaling pathway), and Environmental Information Processing (e.g., bacterial toxins). 

#### 3.2.3. Microbial Community Activity

**Figure 3:**
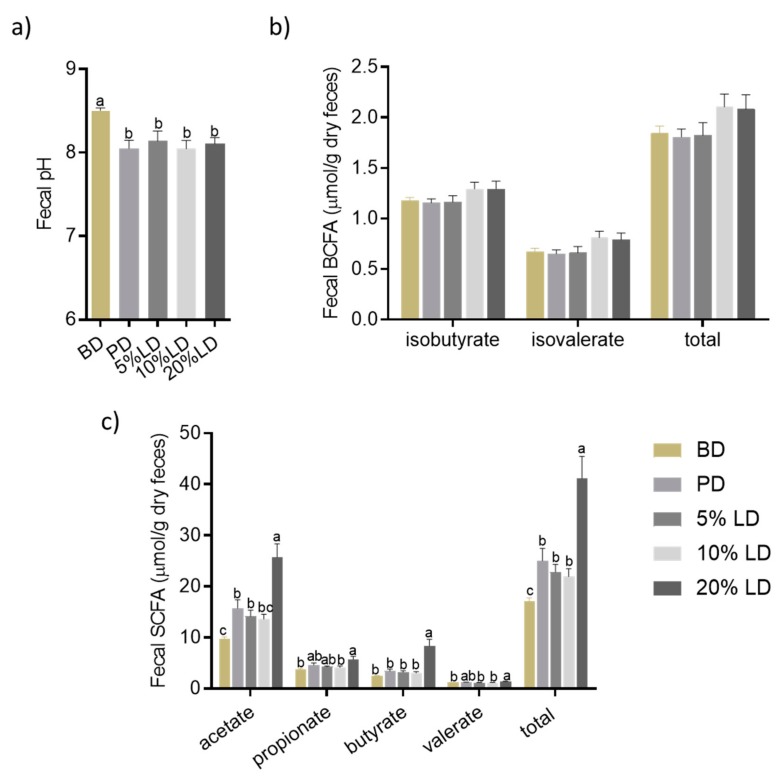
The effects of lentil supplementation on fecal microbial activity. (**a**) Fecal pH; (**b**) fecal branched-chain fatty acids (BCFAs) (µmol/g DW), and (**c**) fecal short-chain fatty acids (SCFAs) (µmol/g DW) of C57bl/6 male mice fed experimental diets for 3 weeks. Values are mean ± SEM; Columns without a common letter differ significantly (*n* = 9/group; *p* < 0.05). BD = basal diet; PD = pectin diet; LD = lentil diet.

Fecal SCFA and BCFA concentrations and pH level were measured as biomarkers of microbial activity. Compared to the BD group, the addition of lentils to the diet resulted in a decrease in fecal pH, independent of lentil supplementation level (Figure 3a). This effect was also observed following consumption of the PD (Figure 3a), suggesting a role of fermentable soluble fiber in the observed effect. While there were no differences observed between dietary groups for fecal BCFA concentrations (products of branched-chain amino acid microbial fermentation) (Figure 3b), fecal SCFA concentrations were enhanced by PD and LD groups compared to BD (Figure 3c). However, mice consuming the 20% LD had the greatest increase in total and individual (acetate and butyrate) SCFAs (Figure 3c), which differed compared to 5 and 10% LD groups. Furthermore, SCFA concentrations following PD consumption were not as high as that observed in the 20% LD group. This may be due to additional fermentable components present in the LD, in particular, GOS and resistant starch [49,50]. 

### 3.3. Consumption of Lentil-Supplemented Diets Alters Colon Barrier Function 

Our previous studies demonstrated that diets supplemented with 20% (*w*/*w*) pulses (e.g., common beans and chickpeas) induced trophic effects on the colonic epithelium, including an increase in crypt length, goblet cell number, and epithelial cell proliferation [15,16,17,22]. As shown in Table 3, the 3-week consumption of either lentil- or pectin-supplemented diets did not alter colon length, weight, epithelial cell proliferation, nor proximal or distal crypt length compared to the BD group. To assess the integrity of the mucus barrier, proximal colon mucus layer thickness was measured. However, there were no differences between dietary groups (Table 3). Furthermore, proximal and distal colon goblet cell density and crypt mucus content (AB score) did not differ between dietary groups (Table 3). In support of these histomorphometrical outcomes, *Muc-1*, *Muc-2* and *Muc-3* mRNA expression did not differ between the groups (Table S1). 

**Figure 4:**
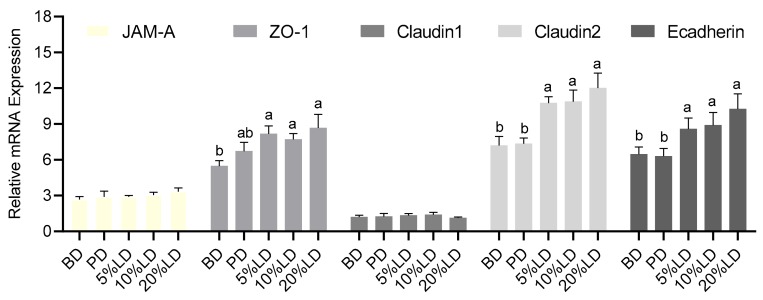
Effect of lentil supplementation on relative mRNA expression of colon apical junctional components. Values are mean ± SEM (*n* = 12/dietary group). Data for each gene were normalized to the expression of the housekeeping gene RPLP0. Bars not sharing a lower-case letter differ (*p* < 0.05). BD = basal diet; PD = pectin diet; LD = lentil diet.

The effects of LDs on biomarkers of epithelial barrier permeability were also assessed in this study. As shown in Figure 4, mRNA expression levels of components of tight and adherens junctional protein complexes were measured. Mice consuming LDs demonstrated increased mRNA expression in the tight junctional component *ZO-1*, pore forming *Claudin-2*, and adherens junctional component E-*cadherin* compared to BD. The pectin-supplemented diet did not alter mRNA expression of these junctional components, suggesting that the effects of lentil were independent of its pectic fiber content. 

**Figure 5:**
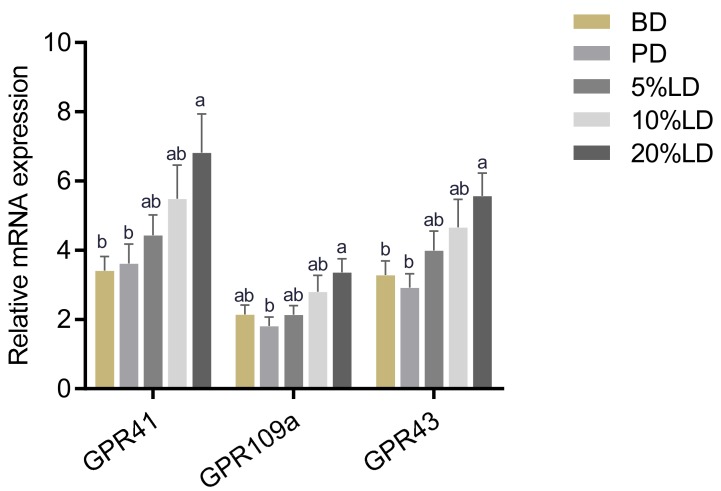
Effect of lentil supplementation on relative mRNA expression of colon SCFA receptors (GPR 41, GPR 43 and GPR 109a). Values are mean ± SEM (*n* = 12/dietary group). Data for each gene were normalized to the expression of the housekeeping gene RPLP0. Bars not sharing a lower-case letter differ (*p* < 0.05). BD = basal diet; PD = pectin diet; LD = lentil diet.

SCFAs are ligands for binding to the orphan G-protein-coupled receptors GPR41, GPR43 and GPR109a. Therefore, their expression levels were assessed in mouse colon tissue [82]. As shown in Figure 5, the 20% LD group showed enhanced GPR41 and GPR43 mRNA expression compared to the BD and PD groups, with the 5 and 10% LD groups having intermediate effects. A similar expression pattern was evident for GPR109a, however, it was only significantly enhanced in the 20% LD group compared to the PD group. 

## 4. Discussion

Recently several countries have adapted their dietary guidelines/food guides to promote consumption of more protein-rich plant foods, including dietary pulses [14,83,84]. Pulses not only serve as protein-rich foods, they are also rich in fermentable non-digestible carbohydrates and various phenolic compounds, which can have beneficial effects on intestinal health. Previously, our group has demonstrated intestinal-health promoting effects of diets supplemented with pulses including common beans (cranberry, kidney, and navy and black bean varieties) and chickpeas [15,16,17]. However, since different pulses vary in their content of bioactives, such as fibre and phytochemicals [5,46,56,85], it cannot be assumed that all pulses will induce similar effects on the intestinal microenvironment. In the current study, we determined for the first time the impact of a diet supplemented with cooked red lentils on structural and functional aspects of the colonic microenvironment in healthy male mice after three weeks of consumption. 

Within the intestinal microenvironment, one of the most pronounced effects induced following the consumption of red lentils was on the microbiota. Diets supplemented with cooked red lentils shifted both the function and the composition of the fecal microbiota (Figure 2, Figure 3, Figure S1). Red lentils increased microbial α-diversity and altered the structure of the microbial community, including a decreased abundance of *Bacteroides* and *Parabacteroides*, whereas the abundance of the SCFA-producing genera, *Prevotella, Roseburia* and *Dorea,* were increased compared to BD [86,87]. The shift in microbial diversity is probably responsible for the significant increase in total fecal SCFAs, as well as acetate and butyrate, following three weeks of red lentil diet consumption (Figure 3). Similar to lentils, diets supplemented with chickpeas, as well as different common beans, increased the abundance of *Prevotella* [15,16]. It is well known that *Prevotella* possess a large spectrum of glycoside hydrolases [88] and their abundance has been associated with high intake of dietary fibre [81,89] and production of SCFAs [90], as well as improved glucose tolerance [91], and lower LDL cholesterol [92], whereas reduced abundance of *Prevotella* has been associated with allergic disease in children [93] and autism [94]. Our results are also in line with previous reports that the genus *Prevotella* abundance is positively associated with a higher intake of plant foods, whereas *Bacteroides* abundance is positively associated with a diet high in animal-based food as well as fat and protein [81]. Further, lentil consumption led to higher abundances of *Roseburia* in mouse feces compared to BD. *Roseburia* is a known-producer of butyrate [87] and decreased abundance of this genus has been associated with several diseases such as Crohn’s disease and colitis [95,96,97]. Hence, consumption of lentils may mediate beneficial effects beyond the promotion of gut health, through mechanisms involving the intestinal microbiota. 

Functional predictions of the metagenome demonstrate enhanced carbohydrate metabolism (Figure S1) supporting the increased SCFA production in the lentil groups. KEGG pathways associated with flavonoid metabolism and biosynthesis were also increased in lentil-fed mice which may be due to the increased level of flavonoids and other phenolic compounds found in lentils (Figure S1) [54,55,56,57,58]. This may indicate that microbial metabolites of lentil flavonoids may participate in the health effects induced following lentil consumption; however, future studies are needed to validate this hypothesis. Furthermore, metagenomes of the BD group were enriched in pathways associated with NOD-like receptor signaling pathway and bacterial toxins, which may indicate a reduced inflammatory tone in the lentil group, which may participate in reducing inflammatory intestinal disorders [98]; however, these predicted functional changes in the metagenome require further investigation. Furthermore, future studies are required to determine the stability of the lentil-induced changes to the structure and function of the microbiota which will help establish the required consumption regime for optimal intestinal health promotion. Studies have shown that dietary changes cause rapid alterations to the microbiota which may indicate that regular pulse food consumption and incorporation into the typical diet may be required for prolonged effects on intestinal health [99,100]. 

To assess the effects of red lentils on colon barrier function, we measured gene expression of tight junction proteins as well as SCFA signaling receptors in colon tissue. Tight junction protein assembly plays a pivotal role in intestinal barrier function [101] and disturbances in tight junction protein expression has been linked to several diseases, such as Crohn’s disease, ulcerative colitis, and celiac disease [102]. Our results indicate that lentil consumption increases the gene expression of ZO-1, Claudin-2, and E-cadherin (Figure 5). It has been shown that other pulses increase the gene expression of tight junction proteins as well, however, the tight junction proteins which are affected differ between pulse types. Chickpeas led to an increased gene expression of ZO-1, junctional adhesion molecule-A (JAM-A) and Claudin-2, whereas beans also affected gene expression Occludin and E-Cadherin, but not Claudin-2 [16,20]. Since this effect was not observed following consumption of pectin-supplemented diet, it suggests that the effects are driven by other lentil components apart from its pectic fibre fraction. *In vitro* studies show that the expression of tight junction proteins can be influenced by polyphenols [103,104], thus polyphenols contained in pulses may mediate the effects on tight junction protein gene expression. These results point to an improved colon barrier function, however, our analysis was limited to gene expression levels, therefore, future studies utilizing functional tests, such as the *in vivo* FITC-dextran or lactulose/mannitol permeability tests, are required to confirm these results. 

Further, SCFAs are ligands for the metabolite-sensing GPRs, *GPR41*, *GPR43* and *GPR 109a* [41]. Similar to the effect observed following navy and black bean consumption [15], mice consuming red lentil diet showed an increase in colonic *GPR41*, *GPR43* and *GPR109a* expression (Figure 5), indicating a higher responsiveness of colonic tissue towards SCFAs. GPR41 and 43 have been shown to play a role in attenuating colitis, in part through regulation of the secretion of inflammatory mediators [105]. Furthermore, butyrate signaling via GPR109a has been shown to induce differentiation of regulatory T cells in the colon, thus playing an important role in maintaining immune homeostasis [106]. 

Similar to the effects we observed with other dietary pulses, consumption of red lentils induced changes to the structure and function of the microbiota (Figure 2 and Figure 3), as well as functional aspects of the intestinal epithelial barrier (Figure 4 and Figure 5). However, in contrast to our previous studies assessing intestinal health effects of other types of pulses [15,16,17,22], red lentil consumption did not enhance architectural changes to the colon barrier, including changes in colon crypt height, goblet cell number, and mucus content (Table 3). Thus, the present study shows that not all pulses exert the same effects on the colonic microenvironment, which may be explained by the varying amounts of bioactive constituents such as dietary fibre and phytochemicals [4,5,6]. In contrast to common beans, lentils contain less dietary fibre, especially lower levels of soluble fibre and GOS which can be fermented by the colonic microbiota into SCFAs [49,50,85]. SCFAs are known to stimulate trophic effects in the colon including crypt elongation, proliferation, and mucus secretion, however, these effects have been shown to be dose dependent [107,108,109] and therefore the observed differences between lentils and other pulse types on the colonic epithelium, may be explained by lower content of fermentable dietary fibre in lentils and lower production of SCFAs, relative to that following consumption of other pulses. In support of this, an *in vitro* fermentation study comparing the degree of fermentability of non-digestible fractions of different pulses and SCFA production, found that fractions from black beans were fermented faster and produced higher levels of SCFAs compared to non-digestible fractions isolated from lentils [38], highlighting that the effects of pulses on aspects of the intestinal microenvironment (e.g. microbial activity) may differ depending on the pulse type. However, future studies are needed to simultaneously compare the effects of different pulse types on intestinal health outcomes *in vivo*, in particular SCFA production.

In this study, we also determined the impact of different physiologically relevant lentil supplementation levels (5–20% (*wt*/*wt*)) on colon health outcomes, thereby increasing the translational potential of this work to human subjects who regularly consume 0.5–2 cups of pulses/day (116–400 g or 58–460 kcals). It has been reported that the highest intake level amongst Canadian pulse consumers is approximately 295 g/day (~1.5 cups or 350 kcal/day, depending on the pulse type), which, as part of a 2300 kcal/day diet, would result in pulse consumption being ~15% of the daily caloric intake [110]. As shown in Table 1**,** 5–20% (*wt*/*wt*) lentil supplementation is equivalent to 4–17% supplementation level by kcal, therefore depending on an individual’s daily caloric intake, this represents 0.5–2 cups of cooked lentils/day. Thus, the supplementation levels used in this study can be considered physiological as they are achieved by pulse consumers. Despite the positive health effects that have been associated with pulse consumption, dietary intake of pulses is low in many countries [111], potentially due to perceived gastrointestinal discomfort [112], lack of knowledge in preparation methods, as well as the inconvenience of long cooking times required for certain dried pulses. Thus, consumer acceptance of pulses could be increased with new pulse ingredients, such as pulse flours and pastes, which are emerging in recent years. Further, increased commercial availability of food products which incorporate pulse ingredients, may help consumers transition to increased consumption of protein-rich plant foods which has recently been recommended in Canada’s new Food Guide.

The results of the present study clearly show that the effects of lentils on the intestinal microenvironment are dose-dependent and the most pronounced effects were observed in the 20% lentil group. For example, this study is the first to show dose-dependent effects of lentils on the microbiota structure, such that the abundance of *Bacteroides*, *Parabacteroides*, and SCFA-producing *Prevotella*, *Roseburia* and *Dorea* were more strongly affected by 20% LD than 5% (Table 2). In line with this, 20% LD had significantly higher SCFA concentrations compared to 5% and 10% lentil groups (Figure 3), and had the highest expression of GPRs in colon tissue (Figure 5). However, the study duration was three weeks and thus further studies are needed to investigate whether these dose-dependent results persist after long-term lentil consumption. 

Finally, we hypothesized that the beneficial effects of lentils may be due, in part, to its soluble fibre fraction (e.g. pectin), therefore we assessed colon health outcomes in mice fed a diet supplemented with 0.7% pectin (*wt*/*wt*); an equivalent level of soluble fibre present in the 20% red lentil diet. Pectins are a complex group of non-digestible polysaccharides, which not only make up the largest portion of the soluble fibre fractions of many fruits and vegetables [113], but are also being studied for their potential use as prebiotics due to beneficial effects on the gut microbiota composition [114,115], fermentability (e.g. SCFA production) [115,116], and anti-inflammatory properties [117]. In this study, 0.7% pectin in the diet induced minimal effects on the intestinal microenvironment, and was clearly less effective than 20% LD. For example, lentil supplementation induced a greater increase in fecal SCFA concentrations compared to pectin diet (Figure 3) and did not increase GPRs gene expression. Similarly, pectin did not influence the abundance of *Bacteroides*, *Dorea* and *Roseburia*, and even though effects were observed between PD and BD for the abundance of *Prevotella* and *Parabacteroides*, the effects were more pronounced in the 20% lentil group. Thus, the results suggest that other substances in lentils such as GOS, resistant starch, and phytochemicals, may also influence the intestinal microenvironment, however, further studies are required to determine which lentil components(s) are driving the specific intestinal health outcomes observed in this study.

In conclusion, supplementation of diets with different levels of red lentils led to beneficial changes in the intestinal microenvironment (some of which were dose-dependent), which have been associated with improvements in intestinal health. In particular, lentils increased microbial diversity and the abundance of SCFA-producing bacteria, especially *Prevotella* leading to increased SCFA concentrations in the feces. Further, colon tissue gene expression of SCFA receptors (GPR41, GPR43 and GPR109) and for tight/adherens junction proteins (ZO-1, Claudin-2 and E-Cadherin) increased, thereby suggesting an improvement in barrier function in the colon. This study also showed that the observed effects are not solely driven by the amount of soluble pectic fibre in the diet, since intestinal health effects induced by pectin supplementation we not always equivalent to those induced by the LD. Overall, this study showed that dietary interventions with cooked red lentils exerted dose-dependent effects on the intestinal microenvironment which can promote intestinal health. Importantly, limitations exist with regards to the translatability of results obtained from murine models to the human situation. Nonetheless, mice are considered important models for human nutrition research [118], in particular for gastrointestinal health, which share important features in anatomy and physiology [119].

## Figures and Tables

**Table 1:** Experimental Diet Composition [g/kg].

Ingredients	BD	PD	5%LD	10%LD	20%LD
Casein	200	200	185	169	138
L-Cystine	3	3	3	3	3
Corn Starch	378	378	352	327	277
Maltodextrin	132	132	132	132	132
Sucrose	100	100	100	100	100
Corn Oil	70	70	69	69	68
Cellulose	70	63	61	52	35
Citrus pectin ^1^	0	7	0	0	0
Mineral Mix	35	35	35	35	35
Vitamin Mix	10	10	10	10	10
Choline Bitartrate	3	3	3	3	3
Lentil Powder ^2^	0	0	50	100	200
Caloric Density (kcal/Kg)	4022	4022	4006.5	4000	3984
Lentil Powder (% kcal)	0	0	4.4	8.7	17.4

^1^ Citrus Pectin from Sigma Aldrich (P9135); ^2^ Cooked lentil powder macronutrient content (% by weight): 27.6% protein; 47.9% carbohydrates; 1.6% fat; 2.7% ash; 2.6% moisture. Cooked lentil powder fiber content (% by weight): 17.6% total fiber; 14.3% insoluble fiber and 3.4% soluble fiber as determined by Maxxam Analytics (Mississauga; Canada); Caloric density: 351 calories/100 g lentil powder; BD = basal diet (AIN-93G); PD = pectin diet; LD = lentil diet.

**Table 2:** Relative abundance of fecal microbial community members (phylum and genus level) in BD-, PD-, 5% lentil-, 10% lentil- or 20% lentil-fed mice.

Taxonomy	BD	PD	5% LD	10% LD	20% LD
**Actinobacteria**	3.25 ± 2.22	1.58 ± 1.48	2.96 ± 3.20	2.08 ± 0.87	1.34 ± 1.20
**Bacteroidetes**	60.80 ± 7.78	47.24 ± 5.80	52.47 ± 8.42	47.36 ± 9.91	47.99 ± 15.23
** Bacteroidaceae (f);** ***Bacteroides***	12.83 ± 3.76 ^a^	13.35 ± 3.94 ^a^	12.10 ± 3.78 ^a^	7.51 ± 3.20 ^b^	7.10 ± 2.50 ^b^
** Porphyromonadaceae (f); Parabacteroides**	4.02 ± 1.34 ^a^	1.35 ± 0.35 ^b.c^	1.88 ± 0.50 ^b^	1.15 ± 0.49 ^c^	0.87 ± 0.31 ^c^
** Prevotellaceae (f);** ***Prevotella***	0.04 ± 0.04 ^a^	0.15 ± 0.17 ^b.c^	0.08 ± 0.05 ^a.b^	0.20 ± 0.14 ^c^	0.67 ± 0.39 ^d^
**Cyanobacteria**	0.33 ± 0.30	0.15 ± 0.18	0.13 ± 0.09	0.14 ± 0.09	0.15 ± 0.10
**Deferribacteres**	0.63 ± 0.55 ^a.b^	1.23 ± 0.57 ^a^	1.00 ± 0.74 ^a.b^	0.82 ± 0.49 ^a.b^	0.34 ± 0.26 ^b^
** Deferribacteraceae (f); *Mucispirillum***	0.63 ± 0.55 ^a.b^	1.23 ± 0.57 ^a^	1.00 ± 0.74 ^a.b^	0.82 ± 0.49 ^a.b^	0.34±0.26 ^b^
**Firmicutes**	33.26 ± 8.73 ^a^	48.85 ± 5.63 ^b^	42.52 ± 9.64 ^a.b^	48.92 ± 9.90 ^b^	48.88 ± 15.71 ^b^
** Clostridiaceae (f);** ***Clostridium***	0.07 ± 0.07^a.b^	0.04 ± 0.03 ^a^	0.19 ± 0.31 ^a.b^	0.15 ± 0.18 ^b^	0.21 ± 0.30 ^b^
** Clostridiaceae (f); Other**	0.10 ± 0.09 ^a^	0.40 ± 0.41 ^b^	0.12 ± 0.15 ^a^	0.11 ± 0.10 ^a^	0.07 ± 0.03 ^a^
** Lachnospiraceae (f);** ***Coprococcus***	0.07 ± 0.04 ^a^	0.75 ± 1.73 ^b^	0.74 ± 0.79 ^b^	0.38 ± 0.23 ^b^	0.38 ± 0.24 ^b^
** Lachnospiraceae (f);** ***Dorea***	0.02 ± 0.01 ^a^	0.03 ± 0.03 ^a^	0.25 ± 0.59 ^b^	0.37 ± 0.44 ^b^	0.43 ± 0.60 ^b^
** Lachnospiraceae (f); Other**	1.66 ± 1.09 ^a^	4.23 ± 1.80 ^b^	2.90 ± 2.19 ^a.b^	3.79 ± 1.91 ^b^	3.34 ± 1.61 ^b^
** Lachnospiraceae (f);** ***Roseburia***	0.00 ± 0.00 ^a^	0.09±0.24^a.b^	0.07 ± 0.06 ^b.c^	0.17 ± 0.11 ^c^	0.25 ± 0.21 ^c^
** Ruminococcaceae (f);** ***Oscillospira***	6.49 ± 1.64 ^a^	9.60 ± 2.31 ^b^	6.76 ± 1.82 ^a^	7.53 ± 1.99 ^a.b^	6.53 ± 1.99 ^a^
** Ruminococcaceae (f);** ***Ruminococcus***	0.81 ± 0.66 ^a^	2.09 ± 2.07 ^b^	2.57 ± 4.03 ^a^	0.83 ± 0.27 ^a.b^	0.71 ± 0.17 ^a^
** Streptococcaceae (f);** ***Lactococcus***	0.14 ± 0.06 ^a^	0.08 ± 0.04 ^a.b^	0.08 ± 0.05 ^b.c^	0.05 ± 0.03 ^b.c^	0.05 ± 0.02 ^c^
** Turicibacteraceae (f);** ***Turicibacter***	1.08 ± 1.09 ^a.b^	0.53 ± 0.63 ^a^	1.18 ± 0.95 ^a.b^	1.70 ± 0.40 ^b.c^	2.19 ± 1.11 ^c^
**Proteobacteria**	0.69 ± 0.42	0.29 ± 0.29	0.39 ± 0.30	0.24 ± 0.07	0.27 ± 0.14
**TM7**	0.08 ± 0.04 ^a^	0.14 ± 0.12 ^a.b^	0.17 ± 0.10 ^b.c^	0.24 ± 0.09 ^c^	0.32 ± 0.24 ^c^
** F16 (f); Other**	0.08 ± 0.04 ^a^	0.14 ± 0.12 ^a.b^	0.17 ± 0.10 ^b.c^	0.24 ± 0.09 ^c^	0.32 ± 0.24 ^c^
**Tenericutes**	0.23 ± 0.24	0.46 ± 0.59	0.22 ± 0.27	0.15 ± 0.12	0.22 ± 0.12
**Verrucomicrobia**	0.67 ± 1.39	0.03 ± 0.04	0.08 ± 0.12	0.01 ± 0.02	0.46 ± 0.72

Displayed are all phyla relative abundances and genera that demonstrated statistical differences between groups (*p* < 0.05 and FDR < 0.05). Values are means ± SD. ^a,b,c^ values in one row without a common letter differ significantly, BD = basal diet; PD = pectin diet; LD = lentil diet.

**Table 3:** Colon histomorphometrics following the 3-week consumption of experimental diets.

Colonic Measurement	BD	PD	5% LD	10% LD	20% LD
**Colon weight [mg]**	22.9 ± 1.1	23.0 ± 1.0	21.0 ± 1.3	23.5 ± 0.9	25.0 ± 1.0
**Colon length [mm]**	75.0 ± 1.7	77.0 ± 1.4	73.6 ± 1.9	76.2 ± 1.4	76.6 ± 1.5
**Proximal crypt length [µm]**	173.1 ± 1.9	159.2 ± 10.9	175.7 ± 4.2	177.2 ± 8.9	176.5 ± 4.7
**Distal crypt length [µm]**	160.2 ± 3.3	156.5 ± 5.7	148.7 ± 3.8	149.7 ± 3.4	151.9 ± 2.8
**Proximal goblet cell #**	21.1 ± 0.7	18.5 ± 1.3	19.7 ± 1.0	22.4 ± 1.0	21.3 ± 0.5
**Distal goblet cell #**	7.0 ± 0.3	6.6 ± 0.3	6.9 ± 0.2	6.5 ± 0.2	6.9 ± 0.2
**Mucus layer thickness [µm]**	24.9 ± 2.7	24.8 ± 2.0	29.4 ± 4.2	33.5 ± 5.1	36.2 ± 4.7
**Mucin content [AB intensity/µm^2^]**	0.43 ± 0.03	0.45 ± 0.03	0.41 ± 0.02	0.45 ± 0.03	0.45 ± 0.03
**Proliferation index**	7.6 ± 0.4	9.1 ± 0.9	11.8 ± 1.3	10.7 ± 1.3	9.6 ± 0.8

Values are means (SEM). The average crypt length was assessed by measuring 15–20 crypts/mouse/location; the number of goblet cells/crypt was assessed by counting goblet cells in 10–15 crypts/mouse/location; mucus layer thickness was measured in the proximal colon with a minimum of 40 measures/mouse; AB-stained colon cross sections were analyzed for colon crypt mucus content. AB = Alcian blue; BD = basal diet; LD = lentil diet; PD = pectin diet.

## Supplementary Materials

The following are available online at https://www.mdpi.com/2072-6643/11/8/1853/s1, Figure S1: Predicted functions of the metagenome, Table S1: Colon mRNA expression in mice fed experimental diets for 3 weeks.

## Abbreviations

AB: alcian blue; BCFA: branched chain fatty acids; BD: basal diet; BW: body weight; EdU: 5-ethynyl-2’-deoxyuridine; FDR: false discovery rate; GOS: galacto-oligosaccharides; GPR: G-protein-coupled receptors; H&E hematoxylin & eosin, IBD: inflammatory bowel disease; LD: lentil diet; OTU: operational taxonomic unit; PCoA: principle coordinate analysis; PD: pectin diet; SCFA: short-chain fatty acid; ZO-1: zona occluden-1
