# Supplementary material for: Cooked Red Lentils Dose-Dependently Modulate the Colonic Microenvironment in Healthy C57Bl/6 Male Mice
Source: Nutrients. 2019 Aug 9;11(8):1853. doi: 10.3390/nu11081853 (PMC6724071; doi:10.3390/nu11081853)
Supplement: Supplementary file 1 [file nutrients-11-01853-s001.pdf]

Supplemental Table S1. Colon mRNA expression in mice fed experimental diets for 3 weeks.[1.]

	BD	PD	5% lentil	10% lentil	20% lentil	p	$p_{linertrend}$
**MUC-1**	5.18 ± 0.82	5.99 ± 1.00	6.12 ± 0.87	7.20 ± 1.46	9.42 ± 1.16	n.s.	0.009
**MUC-2**	1.07 ± 0.17	0.89 ± 0.11	1.15 ± 0.20	1.60 ± 0.37	1.35 ± 0.25	n.s.	n.s.
**MUC-3**	6.10 ± 0.73	5.39 ± 0.68	7.24 ± 0.91	6.24 ± 0.77	6.62 ± 0.78	n.s.	n.s.

[1] Relative abundance of colon mRNA (arbitrary units) in healthy unchallenged mice after 3 weeks of dietary intervention. For each gene, data were normalized to the housekeeping gene RPLP0. Data are means±S.E.M., n=10-12/dietary group. [a,b,c] values in one row without a common letter differ significantly (*P*<0.05).

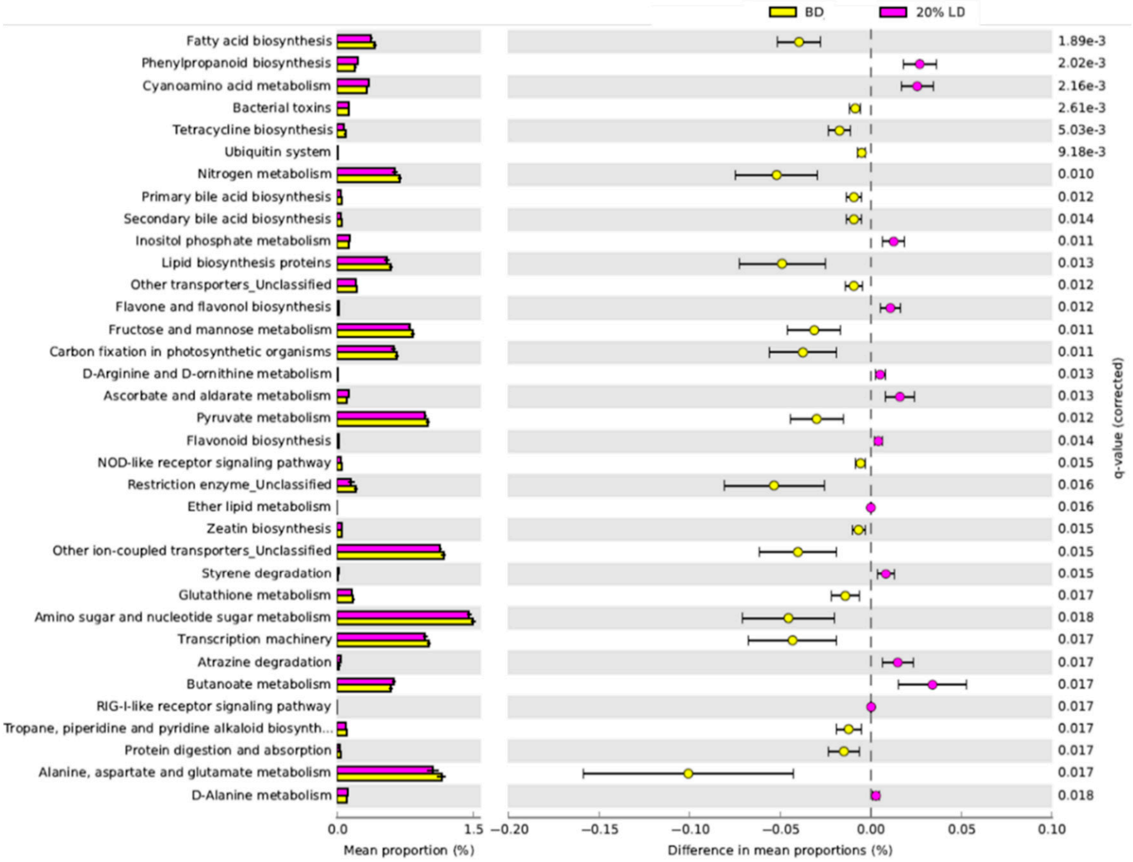

Figure S1. Predicted functions of the metagenome. Functional annotation of the predicted metagenome based on the 16S rRNA data was performed by Phylogenetic Investigation of Communities by Reconstruction of Unobserved States (PICRUSt) and Statistical Analysis of Metagenomic Profiles (STAMP) to identify pathways significantly enriched in the BD group (yellow) or enriched in the 20% LD group (pink). Differences between groups were determined using Welsh's t-test and the Benjamini–Hochberg procedure (FDR corrected p-value (q-value) was set at $p < 0.02$). BD, basal diet; LD, lentil diet.
